# Raised without antibiotics: impact on animal welfare and implications for food policy

**DOI:** 10.1093/tas/txy016

**Published:** 2018-08-10

**Authors:** Joanna Karavolias, Matthew Jude Salois, Kristi T Baker, Kevin Watkins

**Affiliations:** 1 Food and Resource Economics, McCarty A, Gainesville, FL; 2 Elanco Animal Health, Elanco, Greenfield, IN; 3 Elanco Animal Health, Elanco Knowledge Solutions, Greenfield, IN; 4 Elanco, Greenfield, IN

**Keywords:** antibiotics, animal welfare, consumer behavior, food policy

## Abstract

This study assesses the impact of broilers raised without antibiotics and the information gap that exists between consumer perception and production methods. Specifically looking at risk of eye burns, footpad lesions, and airsacculitis, key indicators of animal welfare, bird-level data are collected on the occurrence and severity of each disease state by the type of antibiotic program: no antibiotics ever, nonmedically important antibiotics, or medically important antibiotics. Odds ratios and marginal effects are calculated to understand how the occurrence and severity change with access to medicine. Broilers never given antibiotics had a higher likelihood of disease states investigated, and with greater severity. In some cases, access to nonmedically important ionophores mitigated the risk of occurrence and severity of the conditions. The finding indicates that the growing trend of raising broilers without antibiotics may negatively affect animal welfare. This stands in contrast to existing consumer research showing that consumers purchase poultry raised without antibiotics because they believe that it promotes healthier animals. Therefore, a significant consumer information gap exists which needs to be addressed. JEL Codes: Q130, Q160, Q180

## INTRODUCTION AND MOTIVATION

Poultry raised without antibiotics is becoming more common in grocery stores and restaurants. As new food labels, like no antibiotics ever, become more available to consumers, perceptions of food choice are framed in terms of the label’s quality expectations (Grunert, 2002). The proliferation of labels in food, especially in poultry (e.g., no antibiotics ever, hormone-free, and cage free), has increased rapidly over the years in an effort to differentiate in what is largely a commodity market. Increasingly, consumers are turning to the internet and social media to understand where their food comes from and how it was raised. Although increased access to information through social media outlets allows for easy dissemination of knowledge, it also increases the risk of misinformation reaching consumers (Verbeke, 2005). This has led to an information gap in consumers between perceptions generated by food marketing and actual implications from production practices. If consumers are to make informed decisions when purchasing food, this marketing information gap should be clarified.

**Figure 1.**
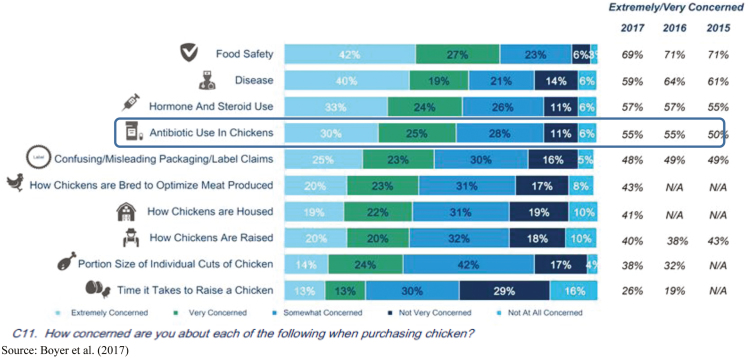
Consumer purchase concerns. Source: Boyer et al. (2017).

**Figure 2.**
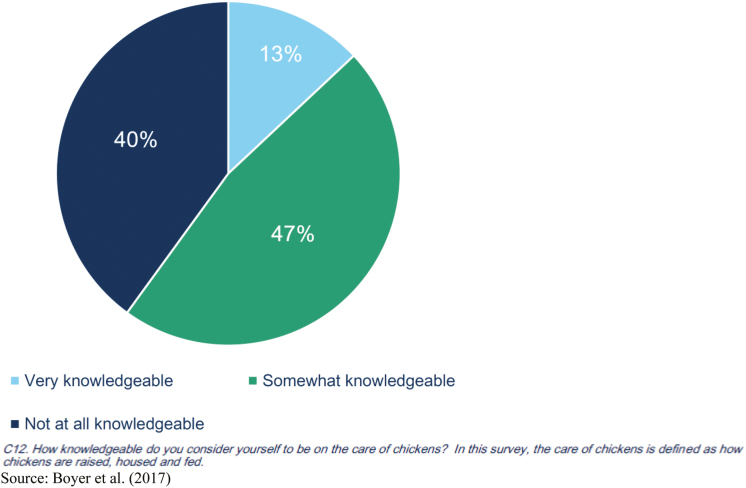
Knowledge level on care of chickens. Source: Boyer et al. (2017).

**Figure 3.**
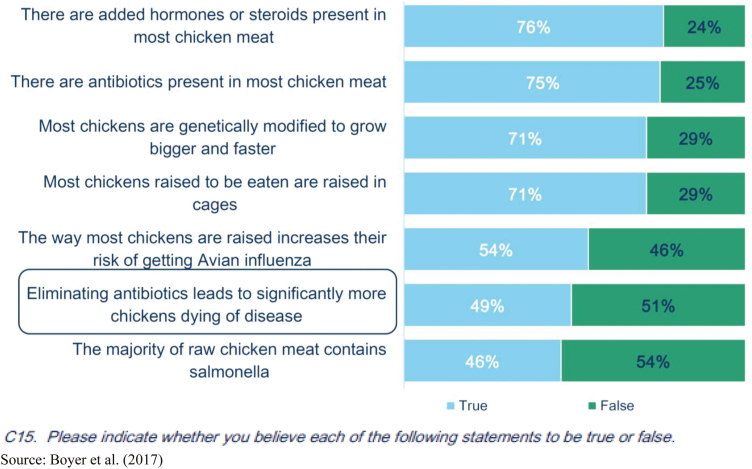
Perceived accuracy of statements about care and raising of chickens. Source: Boyer et al. (2017).

For example, consumer research indicates that antibiotic use in poultry is a top concern when purchasing poultry as depicted in Figure 1 (Boyer et al., 2017). Although these survey results reveal an information gap regarding hormone and steroid use since there are no hormones or steroids used in poultry production, the results also reveal an information gap regarding antibiotic use in poultry. As shown in Figure 2, when questioned about their knowledge level on care of chickens, 60% of consumers believe themselves to be knowledgeable, yet when tested it is revealed that most consumers have incorrect perceptions as described in Figure 3. More than 75% of consumers think that there are added hormones or steroids present in most chicken meat. And less than half acknowledge that eliminating antibiotics can lead to a greater risk of more chickens dying of disease (Smith, 2011; Gaucher et al., 2015).

The information gap is highlighted when examining the prior literature on the impact of raising broilers without antibiotics. These studies generally show an overall negative effect on gut health and bird performance (Smith, 2011; Gaucher et al., 2015). The aim and contribution of this study is to look beyond production performance and assess the impact of eliminating access to antibiotics on measures specifically relating to overall animal welfare. Specifically, we estimate the impact on the occurrence and severity of three important and painful conditions—eye ammonia burns, footpad lesions, and airsacculitis—in live broiler operations that differ in their use of antibiotics. The presence of these conditions is the indication of poor animal welfare with affected birds usually showing reduced weight gain caused by decreased feed intake because of the associated pain. We also extend the analysis beyond raising broilers with no antibiotics vs. medically important antibiotics and look at a third group: broiler raised using nonmedically important antibiotics or ionophores (medicines that have no role in human medicine). As far as we are aware, no study has examined how this specific type of production regime influences animal welfare outcomes. Finally, we use a unique data set sourced from live production operations, which can be more indicative of the true impact from production decisions than in a trial study. Specifically, data are obtained from the Elanco Health Track System, which collects information on bird health from live broiler operations and is used to estimate the impact of restricting access to antibiotics during production on the occurrence and severity of these disease states.

Our findings show that eliminating access to antibiotics can lead to increased risk and severity of specific diseases and that this contradicts consumer perceptions of the elimination of antibiotics supporting good animal welfare. We also find that production using nonmedically important medicines mitigates the potentially negative impact to welfare resulting from antibiotic-free production. From a policy perspective, the decision to purchase poultry never given antibiotics is made with ethical considerations influenced by marketing. No antibiotic ever labels give consumers the choice to take into account environmental and ethical issues when purchasing poultry. Giving consumers the choice to purchase to antibiotic-free poultry does not mean that all consumers will choose to do so; their choice is contingent upon what they value, and their motivation to make use of available information (Grunert et al., 2014). The impact surrounding the no antibiotics ever label, however, is often times unclear to consumers creating a juxtaposition between the ethical framework the consumer believes they are buying into and the reality of the production method. Polices on product labeling and production practices in general may be driving further consumer confusion.

## BACKGROUND AND LITERATURE

### Antibiotic Classification and Use

Antibiotics can be classified into three categories: human-only, animal-only, or shared-class. Human-only antibiotics are only used in people, whereas animal-only antibiotics are only used in animals, such as avilamyacin, and include ionophores. Since ionophores are used against a parasite infection and not bacterial disease, they are similar to many other molecules which are not called antibiotics because they have therapeutic use that is different from an antibacterial. Antibiotics that are not used in human applications and ionophores are considered to be nonmedically important. Shared class antibiotics are antibiotics that are medically important to humans and are also used in animals. In the United States, the Food and Drug Administration has classified antimicrobials as important, highly important, or critically important for human therapy (Appendix A of Guidance 152, 2003). To promote safe and responsible use of medically important antibiotics, the Food and Drug Administration has implemented various marketing status limitations including, prescriptions, and veterinary feed directives to encourage veterinary supervision and safe use.

The United States does not categorize ionophores as medically important antibiotics (FDA, GFI#209, and GFI#152). In the European Union (EU), ionophores are not regulated as antibiotics. Instead, the European Union Regulation (EC) No 1831/2003 categorizes ionophores as “‘coccidiostats’ and ‘histomonostats’ [meaning] substances intended to kill or inhibit protozoa.” The U.S. Department of Agriculture allows meat and poultry processors to label retail meat and poultry as raised without antibiotics and similar statements as long as they can substantiate the statement (FSIS website, 2015). Although ionophores are antimicrobials, they are not regulated as antibiotics by European regulatory agencies, and the European Union allows the use of ionophores when labeling antibiotic-free. Unlike Europe, the U.S. Department of Agricultures’ current interpretation does not allow ionophores to be used with the antibiotic-free label.

Veterinarians and farmers use antibiotics for animals in three ways: 1) To treat animals diagnosed with an illness; 2) To control the spread of illness in a herd or flock; and 3) To prevent illness in healthy animals when exposure is imminent. In addition to these three therapeutic uses, nonmedically important antibiotics are used to also improve production efficiency through a better balance of bacteria for improved nutrition. Animal welfare is related to the use of antibiotics by supporting good health and reducing or eliminating potential pain and suffering associated with disease and/or infection. In particular, controlling and preventing coccidiosis remain a critical concern for any poultry operation.

Coccidiosis is a devastating disease that affects cattle, sheep, goats, swine, and especially poultry. It is caused by microscopic parasites—Coccidia—that inhabit the intestinal tract of animals with mild forms resulting in diarrhea, a consequence of which can reduce growth and limit weight gain (AAF, 2015) to severe forms resulting in concurrent infections with necrotic enteritis and disease progression leading to death (Opengart, 2013). Delaying or avoiding the use of antibiotics can lead to longer time periods of suffering and increase the severity of a disease (Sutherland et al., 2013). Antibiotics are not the only tool, however, used to maintain animal health. Management practices, good nutrition, hygiene, and housing are also very important in supporting good animal welfare. Deprivation of food, water, and bedding, overcrowding, and over-handling increase the incidence of disease in animals and affect animal welfare (Miraglia and Berry, 1962; Tannock and Savage, 1974). The environment the birds are raised in effect the welfare of the animals. Research has shown that the layer hens raised in conventional systems had lower levels of mortality, cannibalism/aggression, and keel damage compared to hens in alternative systems (Coalition for Sustainable Egg Supply, 2015). Although layer hens and broilers are raised in different flocks, the impact of production method, whether it be conventional or an alternative method, is integral for animal health and welfare.

### Consumer Perception of Antibiotics

**Figure 4.**
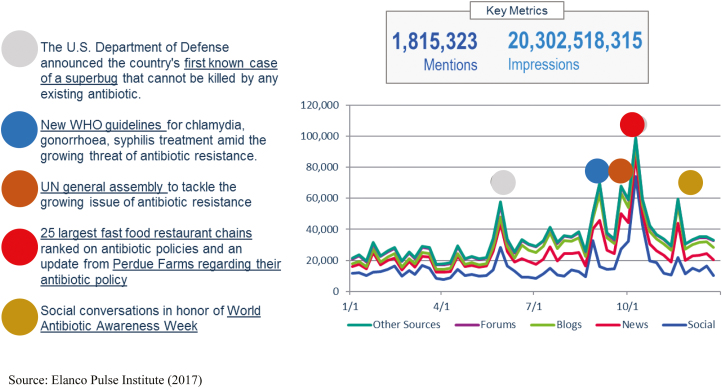
Social media antibiotic conversations overview. Source: Elanco Pulse Institute (2017).

The social media conversation surrounding antibiotics is growing and shifting from government sources to food industry as shown in Figure 4. Globally, the dialogue related to antibiotic use in farm animals increased by 7% in volume in 2016 from 2015 (EPI, 2017). Antibiotic conversations continue to be led by food companies so much so that the top two hashtags in 2016 with more than 45K mentions were #promotion and #perduecrew, coinciding with Perdue’s antibiotic-free marketing campaign. The next highest hashtag was #amr with only 9K mentions (EPI, 2017). “Resistance” was the top term in 2016 by more than 80K mentions, these conversations were driven by multiple events created to bring awareness to antibiotic resistance.

The top Facebook authors by mentions are mainly food companies or food activists, including Subway, Food Babe, and Applegate, applying pressure to reduce or eliminate antibiotic use in their supply chains, often without providing educational content to customers (EPI, 2017). Interestingly, studies, statistics, and research findings contributed greatly to the routine content shared on the topic of antibiotics and farm animals (i.e., farm animals cause of antibiotic-resistant superbug). Headlines focused on the findings of related research most capable of provoking fear among the general populous. Whether accurate or inaccurate, since the antibiotics topic is scientific in nature, social media users are drawn to the research and statistics cited in news articles. These data play an integral role in shaping users’ opinions on antibiotic use as it is easily shareable and, to the general user, credible.

**Figure 5.**
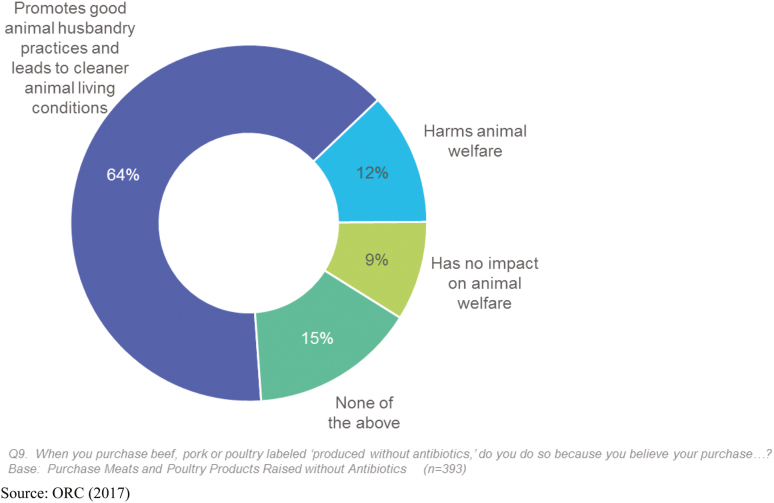
Perceived impact of raised without antibiotics. Source: ORC (2017).

Although it is evident that the use of antibiotics can support good animal welfare through the control, prevention, and treatment of disease, consumers tend to believe that purchasing antibiotic-free poultry perpetuates good animal welfare outcomes. Recent consumer research examined why consumers purchase meats and poultry raised without antibiotics. Of those who purchase meat and poultry products raised without antibiotics, 70% do so because they believe that it is healthier for the animals (ORC, 2017). In addition, nearly two-thirds of those who purchase meat and poultry products raised without antibiotics (64%) believe purchasing beef, pork, and poultry-labeled “produced without antibiotics” promotes good animal husbandry practices and leads to cleaner animal living conditions; far fewer believe that it harms (12%) or has no impact (9%) on animal welfare (Figure 5). This is consistent with the finding in Goddard et al. (2017) where consumers are not aware that banning or restricting antibiotics in livestock production might have negative repercussions for animal welfare.

## DATA AND METHODOLOGY

Data were sourced from the Elanco Health Track System, a proprietary data management system that collected information on over 50 different indicators of general bird health globally since 1993. The data were obtained from participating broiler integrators provided as a service by Elanco Animal Health. Due to the confidential nature of the data, participants cannot be identified, nor can the size of the companies or the locations be disclosed, as the poultry industry would be able to identify those companies. The distribution of companies is the Midwest and East coast for the United States. Admittedly, this does have the potential to bias the sample, but this issue cannot be controlled given the observational nature of the data.

Specific information on bird health was collected via posting sessions (postmortem examinations) conducted by a veterinarian from a subsample of birds representing flocks in production at the grow-out site or barn (note that processing plants are not a part of the dataset). The sample was collected as follows. On average about 5 to 10 healthy birds per flock were sampled at different ages (e.g., average age = X, range = Y, median = Z). They were selected from barns and euthanized solely for the purpose of evaluation for health tracking. Birds were randomly selected from the barn according to an internal protocol that specifies the sampling procedure. The health conditions or lesions tracked were then scored according to a global Elanco Lesion Reference guide, which includes photos of all scores (available upon request). Elanco veterinarians are calibrated on scoring yearly in each region, which reduces the biases between veterinarians, locations, and countries helping us to provide a consistent global database.

Information on the animal health products used during production including antibiotic use was also gathered. The analysis in this paper is based on 2014 bird-level data from the United States. Production was defined by the presence and type of antibiotics used. Birds can be classified in one of the three categories: having no antibiotics or ionophores used at any point during the raising of the bird, although chemical anticoccidials and/or vaccines may have been used, the use of nonmedically important antibiotics, including the use of ionophores and other antibiotics, or the use of medically important antibiotics if shared class or medically important antibiotics were used in production. Other data collected included the date of placement as a chick and the date of the posting session, from which the age of the bird at sampling is determined. Other identifying information collected includes the specific flock and house/barn the bird was sampled from since most producers raise multiple flocks across more than one barn. Confidential information collected includes the genetic breed of the bird, the customer/producer, and geographic locations. To the extent these omitted variables are related to the dependent variable and are correlated with the independent variables, this could introduce omitted variable bias in the coefficient estimates.

Data on eye burns, footpad lesions, and airsacculitis are used to evaluate animal welfare differences in flocks raised without antibiotics compared with those which used antibiotics, both nonmedically and medically important. All three of these conditions are associated with poor gut health and declining conditions of the litter placed on the barn floor to help control dryness and pH levels. As the intestinal health of the bird is compromised, litter becomes saturated with excessive urine and fecal contaminations, elevating the level of ammonia in the litter and risk of bacterial infection, making birds more susceptible to eye burns, footpad lesions, and airsacculitis.

Eye burns cause the eye to be cloudy and can produce ulcerations on the cornea. Since ocular tissue is known to be highly innervated, these ulcerations to the cornea are very painful and result in a reduced state of animal welfare. Levels of pain are also directly correlated to bird health and performance. As broilers stand on litter with increased ammonia levels, the footpads begin to burn causing pain and reduced movement and feeding. The burn site can also be a site of introduction for bacteria causing lameness. Airsacculitis is a respiratory disorder that causes air sacs to be damaged. Airsacculitis can vary from mild to very severe, with more severe cases being a possible indication of a respiratory virus or secondary bacterial infection. Significant airsacculitis can affect overall bird performance, health, morbidity, mortality, and processing ability.

Eye burns were scored on a 0 or 1. A score of zero if the condition was not present or 1 if the bird showed signs of the condition. Footpad lesions and airsacculitis are rated on a 0 to 2 and 0 to 4 scale, respectively. A zero indicates no presence of the condition. As the severity of the condition increases, the rating also increases. Table 1 includes the summary statistics for all three conditions among the three different antibiotic use programs and for the whole sample. The full sample included 11,492 birds, of those 412, 6,054, and 5,030 were never given antibiotics, received nonmedically important antibiotics, or received medically important antibiotics, respectively. Across all three conditions, the mean value is greatest for the raised without antibiotics category indicating a higher average occurrence and severity when various severity levels are present. Similar to the mean, the standard deviation is greatest for raised without antibiotics programs because of the smaller sample size and greater variability in the mean.

**Table 1.** Summary statistics

Condition	Range	No antibiotics ever			Nonmedically important antibiotics			Medically important antibiotics		
		Obs	Mean	SD	Obs	Mean	SD	Obs	Mean	SD
Eye burns	[0,1]	412	0.044	0.205	6,054	0.009	0.094	4.934	0.009	0.093
Footpad lesions	[0–2]	412	0.823	0.620	5,908	0.620	0.742	5,030	0.536	0.716
Airsacculitis	[0–4]	412	0.286	0.658	6,054	0.164	0.500	5,030	0.194	0.547

To estimate the association between production type and the occurrence of a disease, a logistic regression or ordered logit regression is used. Both regressions compute the probability of having a condition as a function of all attributes of other alternatives available (Guadagni and Little, 1983). A logit regression is used for eye burns due to the binary nature of the dependent variable (Equation 1). An ordered logit model is used for both footpad lesions and airsacculitis to account for the varying levels of severity. Both models also accounted for age of the bird in days and the placement defined as a quarterly variable. Both Q1 and Q2 are binary variables (both Q3 and Q4 are omitted dummy variables),

$$\ln\left(\frac{p}{1-p}\right)=\beta_{0}+\beta_{1}RWA+\beta_{2}ANO+\beta_{3}Age+\beta_{4}Q1+\beta_{5}Q2$$ (1)

Odds ratios were calculated using the regression results and have been calculated for the presence of the condition (not accounting for severity). Footpad lesions and airsacculitis were transformed into binary variables: zero if the condition was not observed and one if the condition was observed at any level of severity. Average values were used for the other covariates. The odds ratio is the exponential function of the regression coefficient and represents the constant effect of an antibiotic program on the likelihood that a condition is present in a bird. Marginal effects, which account for severity, were also estimated and measure the change in the predicted probability of a condition occurring as antibiotic use changes. Marginal effects for both the binary and the ordered logit are calculated as a partial derivative with the other covariates held constant at their means. For categorical variables with more than two possible values, the marginal effects show the difference in the predicted probabilities for the cases of one category relative to a reference group. For example, marginal effects will show how the predicted probability of a program that uses medically important antibiotics changes relative to one that does not use antibiotics ever.

## RESULTS AND POLICY IMPLICATIONS

The output summary from the logit regressions is reported in Table 2 for each condition. Across the different models, the coefficient estimates on the no-antibiotics ever indicator are positive and statistically significant. This indicates that the log-odds for each condition occurring is higher for birds raised without antibiotics than a bird raised using medically important antibiotics. The coefficient estimate on nonmedically important antibiotics production method is positive and statistically significant in the footpad lesions model, indicating a similar finding. In the airsacculitis model, the coefficient estimate on nonmedically important antibiotics is negative and statistically significant, indicating that the log-odds of airsacculitis is lower in a bird given nonmedically important antibiotics than one given medically important antibiotics. The other covariates for age and season are statistically significant across the regression models. Age and season all have a significant impact on the presence of eye burns. Older birds are exposed to increased levels of ammonia for longer, likely resulting in an increased chance of eye burns.

**Table 2.** Regression results

Parameter	Eye burns	Footpad lesions	Airsacculitis
Intercept 1	−6.138* (0.379)	−2.089* (0.068)	−4.866* (0.206)
Intercept 2	NA	−0.568* (0.065)	−3.283* (0.123)
Intercept 3	NA	NA	−2.273* (0.104)
Intercept 4	NA	NA	−0.737 (0.095)
No antibiotics ever	1.273* (0.291)	0.372* (0.098)	0.438* (0.132)
Nonmedically important antibiotics	0.253 (0.208)	0.291* (0.038)	−0.228* (0.058)
Age	0.016^†^ (0.009)	−0.001 (0.002)	−0.031* (0.003)
Q1	1.944* (0.249)	0.710* (0.047)	−0.265* (0.074)
Q2	1.235* (0.274)	0.149* (0.046)	−0.499* (0.076)

(Standard error in parentheses).

*Indicates significance at 0.01 level.

^†^Indicates significance at 0.05 level.

The odds ratios for all conditions are reported in Table 3 and provide the odds of a condition occurring when comparing two antibiotic use programs (all odds ratios are significant at the 95% level except for that comparing the presence of eye burns in broilers that received no antibiotics or nonmedically important antibiotics). For example, the odds of eye burns occurring in a bird given no antibiotics is about 3.6 times higher than a bird given medically important antibiotics. The odds of eye burns occurring in a bird raised with no antibiotics is about 2.8 times higher than a bird allowed access to nonmedically important antibiotics. Birds that have been given nonmedically important antibiotics have 1.3 times higher odds of eye burns occurring than a bird given medically important antibiotics. The odds of footpad lesions occurring are higher in broilers never given antibiotics than broilers given medically important antibiotics. The same conclusion holds when comparing production methods that use nonmedically important antibiotics to those which use medically important antibiotics. Since the 95% confidence interval for the odds ratio comparing broilers that received no antibiotics to those animals that receive nonmedically important antibiotics includes one, there is not a statistical difference in the odds of footpad lesions occurring between these two antibiotic programs. Finally, looking the odds ratios for airsacculitis reveals again that the odds of the condition occurring are higher for birds that do not receive antibiotics compared with those that receive nonmedically important or medically important antibiotics. Interestingly, the odds of airsacculitis are lower in animals that have access to only nonmedically important antibiotics compared with animals that received medically important antibiotics.

**Table 3.** Odds ratio for all conditions

Comparison	Eye burns	Footpad lesions	Airsacculitis
No antibiotics ever vs. medically important	3.567 (2.016, 6.305)	1.335 (1.087, 1.252)	1.549 (1.196, 2.005)
No antibiotics ever vs. nonmedically important	2.773 (1.582, 4.863)	0.986 (0.803, 1.211)	1.946 (1.502, 2.523)
Nonmedically important vs. medically important	1.286 (0.855, 1.933)	1.338 (1.252, 1.463)	0.796 (0.711, 0.890)

(95% confidence interval in parentheses).

Marginal effects are presented in Table 4 for eye burns and show the probability of the condition occurring. The sum of all marginal effects is equal to zero; therefore, the marginal effect for a score of 0 is the negative value of the effect presented. There is a 0.022 times greater probability that a raised without antibiotics bird will suffer from eye burns than a bird given medically important antibiotics. Similarly, birds never given antibiotics have a higher probability compared with those given nonmedically important antibiotics. The difference in probability is greater when comparing programs that used medically important antibiotics, implying those programs perform better than programs which use nonmedically important antibiotics when comparing both programs with others that do not use antibiotics. There is no significant difference at the 90%, 95%, or 99% level, between birds in production methods that use nonmedically and medically important antibiotics.

**Table 4.** Marginal effects for eye burns

Severity level	No antibiotics ever vs. medically important	No antibiotics ever vs. nonmedically important	Nonmedically important vs. medically important
1	0.022* (0.006)	0.015* (0.015)	0.003 (0.2170)

*Significance at the 1% level.

*p*-Values reported in parentheses.

Marginal effects are presented in Table 5 for footpad lesions which show how the antibiotic program affects the probability on the severity of the condition occurring. The marginal effects sum to zero which implies the probabilities sum to one (Greene, 1990). There is a lower probability of no footpad lesions occurring, severity level 0, in birds never given antibiotics compared with both other antibiotic programs. The occurrence of no footpad lesions occurring is 0.896 times less likely in birds never given antibiotics compared with those that received medically important antibiotics. In other words, the condition is more likely to occur in a bird that did not receive any antibiotics than a bird that receive medically important antibiotics. For severity levels 1 and 2, the probability of the lesion occurring increases between 0.039 and 0.051 times for raised without antibiotics birds compared with those which were given medically important antibiotics. There is no significant difference between birds not given antibiotics and those given nonmedically important antibiotics.

**Table 5.** Marginal effects for footpad lesions

Severity level	No antibiotics ever vs. medically important	No antibiotics ever vs. nonmedically important	Nonmedically important vs. medically important
0	−0.896* (0.0004)	−0.020 (0.4122)	−0.069* (0.0000)
1	0.039* (0.0000)	0.010 (0.4003)	0.034* (0.000)
2	0.051* (0.0013)	0.011 (0.4229)	0.035* (0.000)

*Significance at the 1% level.

*p*-Values reported in parentheses.

Finally, marginal effects for airsacculitis are presented in Table 6. For all levels of severity, birds never given antibiotics consistently have greater probability of developing the disease. This is true when comparing no antibiotic ever programs with both programs that use antibiotics. Therefore, not only does removing access to antibiotics increase the probability of the condition occurring, but it also increases the probability of more severe cases of the condition as well. When comparing programs that use antibiotics, birds given nonmedically important antibiotics have a lower probability of airsacculitis compared with those given medically important antibiotics at all levels of severity. This result is consistent with the odds ratios reported earlier. One possible explanation of this finding may be the difference in management practices (not measured here) that occurs in production environments in which the use of nonmedically important antibiotics mitigate the risk of airsacculitis (e.g., reduction in bird density in the house).

**Table 6.** Marginal effects for airsacculitis

Severity level	No antibiotics ever vs. medically important	No antibiotics ever vs. nonmedically important	Nonmedically important vs. medically important
0	−0.056* (0.0043)	−0.093* (0.0000)	0.026* (0.0001)
1	0.040* (0.0036)	0.066* (0.0000)	−0.019* (0.0001)
2	0.010* (0.0063)	0.016* (0.0001)	−0.004* (0.0001)
3	0.005* (0.0078)	0.008* (0.0002)	−0.002* (0.0001)
4	0.001* (0.0087)	0.002* (0.0004)	−0.001* (0.0001)

*Significance at the 1% level.

*p*-Values reported in parentheses.

Several limitations to the analysis should be noted. First, results in the analysis represent associations not causality. Second, as already mentioned, given that management practices and other related variables are not tracked by the data, this may introduce a source of omitted variable bias. Transitioning from medically important antibiotics to no antibiotics ever generally requires changes be made to production including reduced stocking density, longer downtime between flock production cycles in a barn, providing an all-vegetarian feed, etc. Third, unobserved differences in animal characteristics might also bias results. For example, animals might receive antibiotics because they are unhealthy. Again, the data cannot address this issue since the antibiotic categories based on the feeding programs listed are predetermined and already in the feed. The data did not track water-based medications or medications that were specifically added during grow-out to address a flock health issue if one came up.

Results are broadly consistent with the few studies that have examined the impact of reducing or eliminating antibiotic use in broiler operations on animal health. Smith (2011) examined the impact of a full drug-free program (no antibiotics or ionophores given at any levels) on overall bird performance and gut health. The author finds that in addition to being more expensive to produce, due to stricter and more expensive diet requirements, drug-free birds had a higher incidence of necrotic enteritis. Smith (2011) highlighted several practices that were found to assist in the control of necrotic enteritis, which include geography, weather, looser density, and a vegetarian diet. Gaucher et al. (2015) conducted a prospective study of 1.55 million birds and evaluated the impact of antibiotic-free conditions on both productivity measures (i.e., livability, condemnations, feed conversion ratio, weight, and density) and cases of necrotic enteritis. Similar to Smith (2011), Gaucher et al. (2015) find that the drug-free program was associated with an overall negative effect on key performance indicators and gut health, which is indicative of the potentially negative effects on the overall animal welfare. In particular, the drug-free program was associated with both an increased incidence of necrotic enteritis, as well as a significant increase in feed conversion, and a decrease in both daily weight gain and mean live weight at slaughter. Our findings take the analysis further and examine specific health conditions that are more indicative of overall broiler welfare beyond measures looking at gut health. Our study uses live production data which can offer useful insights beyond the clinical study conducted in Gaucher et al. (2015). This study also includes a new comparison group relating to the use of nonmedically important antibiotics which was found to mitigate a lot of the negative welfare impact observed in the birds never given antibiotics.

As discussed earlier, consumers believe that poultry that does not receive antibiotics contributes to humane farm practices and good animal welfare outcomes; however, the results in this paper indicate the contrary. Removing access to antibiotics increases the incidence of disease causing painful and harmful living conditions for broilers. Because farm animals are not free of disease risk, in any production setting, important trade-offs need to be understood as producers seek to fill the no-antibiotics ever consumer segment by eliminating access to all antibiotics from farms. Raising broiler flocks without antibiotics may actually increase the potential for adverse animal health and welfare outcomes. Although most producers have substituted improvements in hygiene, vaccinations, and alternative treatments, these changes can be insufficient to adequately address infection risks (Cervantes, 2015). Many of the currently available vaccines do not protect from all coccidian species, leaving gaps in protection as animals develop immune responses. Alternative treatments do not meet the international food safety standards as set by the World Health Organization and Food and Agriculture Organization (Cooper and Singer, 2009; Codex, 2016). Although steps have been taken to provide poultry that has received no antibiotics ever to consumers with little impact on welfare, the complete elimination of antibiotics may not have the intended effects that consumers believe. Hence, there is an information gap with consumers that needs closing.

The results also suggest that a production programs that use nonmedically important antibiotics can mitigate the risks to poor animal health associated with production that eliminates antibiotic use. Rather than perpetuating the idea that poultry can either never be given antibiotics or given medically important antibiotics with no middle ground, information about the use of nonmedically important antibiotics can reduce the confusion associated with poultry labels, while allowing producers’ access to tools to ensure good animal welfare practices (Bowman et al., 2016). It is accepted that antibiotic use is an important factor for determining the development of antibiotic resistance. Antibiotics are societal drugs; each individual use of medically important antibiotics contributes to the sum total of society’s antibiotic exposure (Levy, 1997). However, nonmedically important antibiotics, such as ionophores, do not contribute to antibiotic resistance in humans. Unfortunately, as pointed out earlier, ionophores are classified by the Food and Drug Administration as antibiotics in the United States, unlike in Europe where they are classified as anticoccidials. Therefore, U.S. producers cannot use ionophores if they wish to label their product as no antibiotics ever. Policies that support responsible antibiotic use and allow ionophores to support good animal welfare should be pursued.

There is also an important policy implication with respect to food safety. Loss of broiler health has an impact on broiler cleanliness at the processing plant and can affect food safety. For example, compromised intestinal health may lead to increased food pathogen load due to reduced intestinal strength, which increases leakage/rupture of the intestine during the slaughtering process. Moreover, reduced uniformity in broiler size affected by disease states can increase processing errors like cut or torn intestines due to birds being outside the “calibrated size,” which can result in increased contamination risks (Russell, 2003). Specifically, birds with increased incidence and severity of airsacculitis have been found to have higher contamination rates of *Campylobacter*, *Salmonella*, and *Escherichia coli*—all bacterial species that can harm people and even result in death. The results in this paper suggest that the raising broilers without antibiotics could lead to higher rates of airsacculitis and hence potentially greater risk of bacterial contamination at processing.

## SUMMARY AND CONCLUSIONS

As more poultry growers adopt a raised without antibiotics production program, it is important to understand the consequences to animal welfare and other policy relevant issues, including food safety and consumer perceptions. Prior literature investigating the impact of removing access to antibiotics on poultry production focuses on subtherapeutic (e.g., growth promotion) uses only and/or focuses on productivity impact related to bird performance and grower financial outcomes (Emborg et al., 2001; Engster et al., 2002; Graham et al., 2007; MacDonald and Wang, 2011). This study examines the impact of three different antibiotic access programs (no antibiotics ever, nonmedically important antibiotics, and medically important antibiotics) covering therapeutic uses on the risk and severity of three health conditions: eye burns, footpad lesions, and airsacculitis. These conditions are indicative of broiler welfare due to disrupted biological functions and the negative experience of pain associated with certain disease states. Using proprietary industry data from the Elanco Health Tracking Study, econometric estimates on the likelihood of the conditions occurring across levels of severity are estimated. Findings reveal that broilers raised without antibiotics have a greater incidence of eye burns, footpad lesions, and airsacculitis occurring, and occurring more severely, compared with broilers given either nonmedically important or medically important antibiotics. The risk is mitigated to some extent by programs that allow access to nonmedically important antibiotics including ionophores.

These findings have important policy implications. First, the results here would seem to indicate a significant information gap existing with the consumer on the topic of access to antibiotics on the farm. Available consumer research demonstrates that consumers purchase poultry never given antibiotics because they believe it is better for the animal (ORC, 2017). As shown in this paper, removing access to antibiotics increases the risk and severity across three diseases states known to reduce animal welfare. Studies that investigate consumer willingness to pay for food produced from animals never given antibiotics, as well as the human-animal tradeoffs from policies restricting antibiotics access (e.g., McNamara and Miller (2002), Secchi and Babcock (2002), and Lusk et al. (2006)) should include impact on animal welfare in the analysis and include therapeutic uses (i.e., control, prevention, and treatment).

Second, current U.S. policies do not allow ionophores—a class of nonmedically important antibiotics that have no role in human medicine and are not known to affect risk of antimicrobial resistance in humans—to be used under a program that never uses antibiotics. Although European authorities do not classify ionophores as antibiotics, the U.S. policy reduces the capacity of farmers and veterinarians to address disease risks in an antibiotic-free production environment. Lastly, there is an important connection with animal welfare and human health through food safety. For example, Volkova et al. (2013) have shown that an anticoccidial program may improve control on *Salmonella* contamination of poultry carcasses and *Campylobacter* contamination at the farm level. In addition, Russell (2003) found that chickens with airsacculitis have lower weights, more fecal contaminations, more processing errors, and higher levels of contamination of bacteria such as *Campylobacter*. These findings are important from a food safety perspective as the U.S. Centers for Disease control estimates that *Campylobacter* causes 1.3 million infections, 13,000 hospitalizations, and 120 deaths each year in the United States (CDC, 2013).

Policies aimed at eliminating or restricting the use of antibiotics in broiler production may come with potentially negative consequences with respect to good animal welfare. A more effective policy approach should consider comprehensive animal care plans that incorporate good housing, management, and responsible antibiotic use, including the use of ionophores. Policies aimed at informing the consumer on the positive role of access to antibiotics in supporting good animal welfare while limiting risk of antibiotic resistance in humans are needed to address the current information gap.
